# Anti-acne vulgaris effect including skin barrier improvement and 5α-reductase inhibition by tellimagrandin I from *Carpinus tschonoskii*

**DOI:** 10.1186/s12906-019-2734-y

**Published:** 2019-11-21

**Authors:** Jun Yin, In Hyoek Hwang, Min Won Lee

**Affiliations:** Laboratory of Pharmacognosy and Natural Product derived Medicine, College of Pharmacy, Chung-Ang University, Seoul, 156-756 Republic of Korea

**Keywords:** *Carpinus tschonoskii*, 5α-reductase inhibition activity, Anti-acne vulgaris, Skin barrier, Anti-inflammation, Anti-oxidant activity

## Abstract

**Background:**

*Carpinus tschonoskii* (CT) has been previously studied for various activities in the improvement of skin diseases. In the present study, we examined the in vitro anti-acne vulgaris (AV) effect of CT leaves (CTL) and tellimagrandin I (TI), one of the main ellagitannins from CT, including skin barrier improvement and 5α-reductase inhibitory activity.

**Methods:**

To test the anti-AV activities of CTL and TI, firstly, anti-oxidative and anti-inflammatory activities including DPPH radical scavenging activity, nitric oxide (NO) inhibitory activity, and cytokines [interleukin (IL)-6 and IL-8] were tested. Skin barrier improvement experiments were tested using developing cornified envelope (CE) formation, and filaggrin mRNA expression level was determined by RT-PCR. The 5α-reductase inhibitory activity was determined by measuring the testosterone levels in rat liver microsomes.

**Results:**

CTL and TI showed potent anti-oxidative activity and anti-inflammatory activities. Especially, the cytokine production inhibitory activities of TI were found to be similar to the positive control, epigallocatechin gallate (EGCG). CTL and TI enhanced the CE formation and filaggrin mRNA expression levels and showed potent activities compared to that in the positive control, 1.5 mM Ca^2+^. In additionally, CTL and TI showed 5α-reductase inhibitory activities in a dose-dependent manner.

**Conclusion:**

The results showed that CTL and TI inhibit AV endogenous factors such as 5α-reductase and inflammatory cytokines and affect exogenous factors such as developing skin barrier function (CE and filaggrin levels). Therefore, CTL and TI may be plant-derived agent, promising in the treatment of acne vulgaris.

## Background

The skin confers protection from external stimuli including physical and chemical stresses such as air pollution, ultraviolet radiation, and wounds. Differentiation of keratinocytes is essential to maintain the barrier function of skin [1, 2]. Keratinocytes have a role in the skin barrier function and proliferate in the basal layer, being increasingly differentiated from the spinous layer to corneocytes. In the terminal differentiation process, increase of intercellular Ca^2+^ levels induces expression of periplakin, involucrin, loricrin, filament aggregating protein (filaggrin), and other factors that promote differentiation. In particular, filaggrin plays an important role in the formation of a cornified envelope (CE), because it binds to and is responsible for aggregation of keratin filaments. These skin barrier factors crosslink keratin filaments and ceramide in the plasma membrane, resulting in the formation of a CE, which is a strong physical barrier and permeation barrier comprising 10 nm thick layers of insoluble proteins [3–6].

When keratinocytes are exposed to external stimuli, they produce inflammation-induced factors including free radicals, cytokines, and nitric oxide (NO), resulting in inflammatory reactions. These inflammatory reactions impair the skin barrier [4, 6–8]. Keratinocytes produce a variety of cytokines that regulate cellular communication. Deregulated cytokine expression can thus contribute to a dysfunctional skin barrier as observed in chronic inflammation disease [8–10]. The cytoplasm of keratinocytes in all epidermal layers contains the pro-inflammatory cytokines, interleukin-6 (IL-6), IL-8, and tumor necrosis factor α (TNF-α) Especially, IL-6 is reported to downregulate filaggrin [8, 10–17]. Additionally, free radicals including reactive oxygen species (ROS) mediate oxidative stress and produce inflammatory cytokines such as IL and TNF contribute to the pathogenesis of many inflammatory skin diseases [8, 10, 18].

Acne vulgaris (AV) is a chronic inflammatory disease of the pilosebaceous units resulting from androgen-induced sebum production and inflammation [19–22]. Among these causes, androgen-induced sebum production is associated with 5α-reductase reactions [23–25]. The enzyme 5α-reductase metabolizes testosterone to dihydrotestosterone (DHT). However, imbalance between testosterone and DHT causes acne and benign prostatic hyperplasia [26, 27]. In AV, sebum secretion, dehydration of the corneum, and transepidermal water loss (TEWL) are increased. In clinical research, TEWL is an important factor for skin barrier function. A marked increase in TEWL was observed in patients with AV of moderate severity compared to that in patients with AV of mild severity and in normal controls. These results indicate that the degree of corneum permeability barrier impairment directly matches the severity of AV. In addition, the follicular epithelium also contributes to skin barrier functions. Impairment of the skin barrier occurs when the intensity of inflammation increases and weakens the follicular wall [28, 29].

*Carpinus tschonoskii* (CT), a deciduous broad-leaved arboreous tree, is a member of the genus *Carpinus* and Betulaceae family, native to Korea, Japan, and China [30, 31]. The genus *Carpinus* has been widely and traditionally used to treat bladder infection, osteoporosis, and anxiety disorders [32]. According to the “Coloured flora of Korea,” leaves of CT are oval shaped and have a doubly serrate margin [33]. The bark of CT has been used as a material for furniture and bed logs [34]. In a previous study, CT has been confirmed to show biological activities including cytoprotective activities, suppression of tyrosinase expression, whitening activities, anti-wrinkle, anti-allergic activities, and neuroprotective activities [31, 34–36].

Despite many studies on skin diseases, there have been few experiments demonstrating the skin improvement effects of CT in AV. The purpose of this study was conducted to assess the skin improvement effects of CT leaf (CTL) extract and tellimagrandin I (TI), which was isolated from CTL, on AV.

## Methods

### Plant materials

The leaves of *Carpinus tschonoskii* were obtained from the Yeoju Eco Park, Yeoju, Republic of Korea, in July 2018. Plant materials were distinguished by Kim Sungsik, Ph.D. (Korea National Arboretum, Pocheon). Voucher specimens were placed at the herbarium of the College of Pharmacy, Chung-Ang University (CTLYZ-1806).

### General experimental procedures

The column chromatography isolation was performed on a Sephadex LH-20 column (10–25 m; GE Healthcare Bio-Science AB, Uppsala, Sweden). Structural identification was by one-dimensional nuclear magnetic resonance (1D-NMR) including ^1^H-NMR (600 MHz) and ^13^C-NMR (125 MHz) (JEOL, Tokyo, Japan) at Chung-Ang University.

### Extraction, isolation and structure elucidation

Leaves (1.2 kg) of CT were extracted for 72 h at room temperature (25 °C) with 70% prethanol A (ethyl alcohol), after removing the solution under vacuum, the CTL extract (252 g) was obtained. The CTL extract (142 g) was dissolved in water, the water layer was filtered through Celite 545 (Duksan Pure Chemicals Co. Ltd., Seoul, Korea). Filtrate was concentrated and applied to Sephadex LH-20 column (25–100 μm; Pharmacia, Uppsala, Sweden) and eluted with water (H_2_O)-methanol (MeOH) gradient system, eleven fractions were obtained. Repeated column chromatography of fraction 10 (11.64 g) on Sephadex LH-20 with water-methanol gradient system to obtained tellimagrandin I (TI, 1.98 g). The structure of TI was identified by analysis of 1H-NMR and 13C-NMR spectra and comparison with reference [31].

### Chemical and reagents

Dulbecco’s Modified Eagle Medium (DMEM), trypsin, and fetal bovine serum (FBS) were purchased from Welgene (Gyeongsan, Republic of Korea). Streptomycin–penicillin was purchased from Gibco (NY, USA). Calcium-free DMEM, superscript™ IV first-strand synthesis system, and Dream taq Green PCR Mix were purchased from Thermo Fisher Scientific (MA, USA). TRIzol reagent was purchased from Invitrogen (CA, USA). Sodium dodecyl sulfate (SDS), dithiothreitol (DTT), agarose, lipopolysaccharide (LPS), ethyl ether, 1,1-diphenyl-2-picrylhydrazyl (DPPH), Griess reagent, NG-Methyl-l-arginine acetate salt (L-NMMA), and thiazolyl blue tetrazolium bromide (MTT) were obtained from Sigma Aldrich (St. Louis, USA). Reagent set B, cytokine IL-6 and IL-8 ELISA sets used for immunoassay were purchased from BD Biosciences (NJ, USA). TI was acquired in a previous study [31].

### Anti-oxidative activity

#### Measurement of DPPH radical scavenging activity

To evaluate the radical scavenging activities of CTL extract and TI, DPPH assay was conducted. DPPH is bound with the hydrogen of anti-oxidants, because nitrogen in hydrazyl on DPPH has an unstable radical. DPPH radical scavenging activities were assessed by confirming the color change in DPPH accompanied with the reaction to anti-oxidants [37]. To assess anti-oxidant activities, samples dissolved in anhydrous ethyl alcohol were added (20 μL) into a 96-well plate, followed by addition of 0.2 mM DPPH (180 μL). No sample adding, 0.2 mM DPPH 200 μL was made as the negative control. After gentle shaking for 15 min at room temperature, optical density (OD) was measured at 517 nm using an ELISA reader (TECAN, Salzburg, Austria). The OD was used for calculation as follows: the rate of inhibition (%) = [1 - (sample OD/negative control OD)] × 100. IC_50_ values were defined as the concentration needed to scavenge 50% free radicals. Ascorbic acid served as the positive control [38].

#### Cell culture

RAW 264.7 cells were purchased from the Korean Cell Line Bank. HaCaT cells (spontaneously immortalized keratinocyte cell line) were purchased from the Dermatology department of Chung-Ang University hospital. These cells were incubated at 37 °C in a humidified atmosphere (5% CO_2_) in DMEM containing 10% FBS, 100 IU mL^− 1^ penicillin G and 100 mg mL^− 1^ streptomycin.

#### Cytotoxicity

To evaluate the cytotoxicity of the CTL extract and TI, MTT assay was performed, and absorbance resulting from the mitochondrial conversion of MTT to formazan was measured. MTT is the colorimetric assay used to measure cell proliferation, viability or cytotoxicity. Cells were inoculated into a 96-well plate and placed in the incubator for 6 h. The cells were then treated with samples at various concentrations (12.5, 25, 50, and 100 μg mL^− 1^ or μM) and incubated for an additional 24 h. Afterward, the medium was replaced with 100 μL of phosphate buffered saline (PBS) containing 0.5 mg mL^− 1^ MTT and incubated for a further 4 h at 37 °C. MTT-PBS was then removed, and the formazan was dissolved in 100 μL dimethyl sulfoxide. The extent of MTT reduction to formazan within the cells was quantified by measuring the absorbance at 540 nm by using an ELISA reader. The cytotoxicity was calculated as cell viability (%) = sample OD/blank OD × 100.

### Anti-inflammatory activity

#### Measurement of inhibitory activity on NO production

RAW 264.7 cells were inoculated into a 96-well plate and incubated for 6 h at 37 °C in 5% CO_2_. The cells were then incubated in a medium containing 1 μg mL^− 1^ LPS and the samples. After incubating for an additional 24 h with samples (100, 50, 25 μg mL^− 1^ for extract level; 100, 50, 25 μM for compound level), the NO contents were evaluated by Griess assay. Griess reagent was added to each of the supernatants from the cells. L-NMMA was used as a positive control. Inhibition of NO synthesis was calculated as inhibition rate (%) = [1 - (sample OD - blank OD)/(control OD - blank OD)] × 100, and the IC50 values were defined as concentrations that inhibit 50% of NO production [38].

#### Measurement of inhibitory activity on cytokine production

Differentiated HaCaT cells were inoculated into a 96-well plate and placed for 6 h at 37 °C in 5% CO_2_. A medium containing 1 μg mL^− 1^ LPS and samples were added to the wells and incubated for an additional 24 h. Afterward, the supernatant from the cells was stored in a 1.5 mL tube [39]. Concentrations of cytokines IL-6 and IL-8 were analyzed in the culture supernatants by ELISA following the manufacturer’s protocol. Cytokine production was measuring using the ELISA reader at 450 nm. The results were calculated using a standard calibration curve [40].

#### 5α-reductase inhibitory activity

Liver microsomes containing 5α-reductase from 7-week-old male Sprague-Dawley (SD) rats were prepared. (Han Lym Lab. Animal Co., Hwaseong, Korea) Mature male SD rats were anesthetized using ethyl ether. Using a strong pair of dissecting scissors, the chest cavity of SD rats was opened and the hepatic portal vein was nicked. A syringe was used to inject cold STM buffer (270 mM sucrose, 10 mM Tris-HCl, pH 7.5, and 1 mM MgCl_2_, G-BIOSCIENCES, MO, USA) into the left ventricle of the heart. STM buffer was thus perfused to the liver and the color of the liver changed from a dark red to a light pink. The liver was then quickly dissected and minced in a beaker by using a pair of scissors. The minced tissue was then homogenized in 3-tissue volumes of STM buffer [41]. The homogenates were centrifuged at 10,000×*g* for 10 min. The pellet was washed with 2-pellet volumes of STM buffer. The supernatant from the centrifugation contained microsomes suspended in STM buffer; these were homogenized using a syringe with 18 G, 23 G, and 25 G needles [42]. The microsomes were divided into aliquots and stored in a deep freezer (− 80 °C); before use, the microsomes were diluted to 1/5 concentration.

To evaluate the inhibitory activity on 5α-reductase, each group was treated with phosphate buffer (pH 6.5), testosterone (500 μg mL^− 1^, 100 μL), microsomes (700 μL), nicotinamide adenine dinucleotide phosphate (NADPH, 770 μg mL^− 1^, 350 μL), and the sample or finasteride (600 μL) except the intact group and normal group. Finasteride was used as a positive control, which led to a decrease in DHT concentration. The intact group was the enzyme blank group, and the normal group had the full activity of 5α-reductase reaction without interference. The reaction was carried out in the incubator for 30 min at 37 °C. To complete the reaction, 2 mL of dichloromethane was added to every group [43]. All groups were stored at − 25 °C and concentrated to obtain an unfrozen organic solvent layer. The concentrates were dissolved in 500 μL of 50% methanol. Testosterone was quantified using HPLC instead of radioimmunoassay (RIA), because the RIA method has hazards from radioactive compounds and requires complex equipment. The analyte was eluted at a flow rate of 1 mL min^− 1^ using the binary gradient H_2_O (A) and ACN (B). The quantification wavelength of these chromatograms was set at 242 nm, which was optimized for testosterone. The injection volume was 10 μL. The data were analyzed using the Autochro-Win 3.0 plus software system. The following equation was used for calculation: 5α-reductase inhibitory rate (%) = [1 - (area of sample – area of intact)/(area of normal – area of intact)] × 100, and the IC_50_ values were defined as the concentrations that inhibit 50% of the 5α-reductase reaction.

#### mRNA expression of filaggrin

HaCaT cells were incubated at 37 °C in 5% CO_2_ in Ca^2+^ free DMEM containing 10% FBS, 100 IU mL^− 1^ penicillin G, 1.5 mM CaCl_2_, 1 M HEPES, and Glutamax. HaCaT cells were inoculated into 6-well plates at 3 × 10^5^ cells mL^− 1^ and incubated in a 5% CO_2_ incubator at 37 °C for 24 h. The medium was then removed, and the cells were treated with appropriately diluted CTL extract and TI along with 20 ng mL^− 1^ TNF-α. The treated cells were incubated for about 1 day [17, 44].

Total RNA was extracted using TRIzol reagent. Next, 0.5 μg of total RNA was reverse-transcribed to cDNA using a Superscript™ IV first-strand synthesis system. The cDNA was used as a template for PCR amplification with 22 cycles using a DNA engine gradient cycler (MJ Research, Inc., MA, USA). The cycles consisted of the following steps: denaturation for 30 s at 95 °C, annealing at 56 °C for 30 s, and extension at 72 °C for 1 min [4]. β-Actin was used as the internal control. The PCR primers used were as follows: filaggrin, sense, AAGCTTCATGGTGATGCGAC, antisense, TCAAGCAGAAGAGGAAGGCA; and β-actin, sense, ACACTGTGCCCATCTACGAGGGG; antisense, ATGATGGAGTTGAAGGTAGTTTCGTGGAT.

The bands were visualized using a chemiluminescence imaging system (Fusion Sl, Collégien, France). Expression of filaggrin mRNA was normalized to that of β-actin by densitometry, and the EC_50_ values were defined as concentrations that caused a 50% effect.

#### Cornified envelope formation

CE assay was performed as an experimental method to measure keratinocyte differentiation ability. HaCaT cells were inoculated into 6-well plates at 3 × 10^5^ cells mL^− 1^ and cultured in a 5% CO_2_ incubator at 37 °C for 24 h. Following the culture, the medium was removed and the cells were treated with required dilutions of CTL extract and TI in a medium containing 0.05 mM CaCl_2_. The normal control was treated with medium containing 0.05 mM CaCl_2_ and the positive control was treated with medium containing 1.5 mM CaCl_2_. After treatment with the samples, the cells were incubated for about 5 days.

The cells were then harvested and 1 mL of 10 mM Tris-HCl (pH 7.4) containing 2% SDS and 20 mM DTT was added to the pellet containing the cells. After this, the supernatant was sonicated for 3 min and boiled at 100 °C for 10 min. The samples were centrifuged (1200 rpm, 30 min, 4 °C), the precipitates were suspended in PBS, and the absorbance was measured at 340 nm. The following equation was used for calculation: CE formation rate (%) = [1 - (sample OD - normal OD)/(control OD – normal OD)] × 100, and EC_50_ values were defined as the concentrations that cause a 50% effect on CE formation [44].

### Statistical analysis

Analysis of variance was performed using SPSS software (SPSS, Inc., Chicago, IL, USA). All data are expressed as the mean ± SD of triplicate experiments, and statistically significant differences were analyzed by one-way analysis of variance (*p* < 0.05), and t-test (***, *p* < 0.001; **, *p* < 0.01; *, *p* < 0.05).

## Results

### Anti-oxidative activity

#### DPPH radical scavenging activity

To assess the anti-oxidative activities of CTL extract and TI, DPPH radical scavenging activities were measured. CTL extract (IC_50_ = 31.42 ± 0.73 μg mL^− 1^) showed potent DPPH radical scavenging activities compared with the positive control, ascorbic acid (IC_50_ = 14.67 ± 2.90 μg mL^− 1^). TI (IC_50_ = 12.88 ± 0.30 μM) also showed potent DPPH radical scavenging activities compared with ascorbic acid (IC_50_ = 9.13 ± 0.49 μM) (Table 1).

**Table 1:** IC_50_ values of *Carpinus tschonoskii* leaf (CTL) extract and tellimagrandin I (TI) on each bioactivity, including free radical scavenging activity, inhibitory activity of NO production, IL-6, IL-8 and 5α-reductase

Samples		IC_50_				
		radical scavenging activity	inhibitory activity of NO	inhibitory activity of IL-6	inhibitory activity of IL-8	inhibitory activity on 5α-reductase
(μg mL^−1^)	CTL	31.42 ± 0.73^a^	5.98 ± 0.33^a^	14.20 ± 7.07^a^	7.46 ± 2.60^a^	197.81 ± 1.92^a^
	Positive Control	14.67 ± 2.90^b^	1.48 ± 0.98^b^	2.98 ± 1.47^b^	0.74 ± 0.09^b^	40.65 ± 9.56^b^
Compound (μM)	TI	12.88 ± 0.30^a^	5.44 ± 1.84^a, b^	6.82 ± 2.37^a^	0.56 ± 0.56^a^	331.75 ± 14.62^a^
	Positive Control	9.13 ± 0.49^b^	2.35 ± 1.14^b^	6.68 ± 1.86^a^	0.56 ± 0.52^a^	45.98 ± 15.40^b^

The results were expressed as mean ± standard deviation (SD) of triplicate experiments. (*n* = 3) ^a-b^in the same column are significantly different, *p* < 0.05. Positive control of free radical scavenging experiment is ascorbic acid, positive control of inhibitor activity of NO production is L-NMMA, positive control of inhibitory activity of IL-6 and IL-8 is EGCG, positive control of inhibitory activity of 5α-reductase is Finasteride

#### Cytotoxic activity

Before evaluating the improvement effects on skin barrier and AV, MTT assay was conducted to determine the cytotoxicity of CTL extract and TI on RAW 264.7 cells and HaCaT cells. No cytotoxicity of these compounds was observed (data not shown).

### Anti-inflammatory activity

#### Inhibitory activity on NO production

In order to assess the anti-inflammatory activities of CTL extract and TI, NO inhibitory activities were measured in RAW 246.7 cells. As shown in Table 1, CTL (IC_50_ = 5.98 ± 0.33 μg mL^− 1^) extract showed potent anti-inflammatory activity, and TI (IC_50_ = 5.44 ± 1.84 μM) showed potent inhibitory effects against NO production compared with the positive control, L-NMMA (IC_50_ = 2.35 ± 1.14 μM).

#### Inhibitory activity on cytokine production

To assess the inhibitory effects on IL-6 of production, cells were treated with LPS (1 μg mL^− 1^) to induce inflammation. After LPS exposure, the inhibitory effects of CTL extract and TI on the cytokine levels was measured. IL-6 concentration was decreased in the sample-treated groups. As shown in Table 1, CTL extract (IC_50_ = 14.20 ± 7.07 μg mL^− 1^) showed potent anti-inflammatory activity. TI (IC_50_ = 6.82 ± 2.37 μM) showed a similar inhibitory activity compared with the positive control, EGCG (IC_50_ = 6.68 ± 1.86 μM) against IL-6 production.

These inflammatory reactions result in impairment of the skin barrier. As shown in Table 1, CTL extract (IC_50_ = 7.46 ± 2.60 μg mL^− 1^) showed moderate IL-8 production inhibitory activity. TI (IC_50_ = 0.56 ± 0.56 μM) also showed an equal inhibitory activity compared with the positive control, EGCG (IC_50_ = 0.56 ± 0.52 μM) against IL-8 production.

#### Cornified envelope formation activity

Relative CE formation levels were evaluated based on the normal control, the 0.05 mM Ca^2+^ treated group. CE formation levels showed potent increases with the CTL extract compared with the positive control (Table 2). At the compound level, the CE formation level showed potent increases with TI compared with the positive control, with 100 μM of TI showing strong CE formation.

**Table 2:** EC_50_ values of *Carpinus tschonoskii* leaf (CTL) extract and tellimagrandin I (TI) for effective activity on cornified envelope (CE) formation

Extract	IC_50_ (μg mL^−1^)	Compounds	IC_50_ (μM)
CTL	30.42 ± 4.79	TI	27.15 ± 3.24

The results are expressed as mean ± standard deviation (SD) of triplicate experiments. (*n* = 3)

#### mRNA expression of filaggrin

**Fig. 1:**
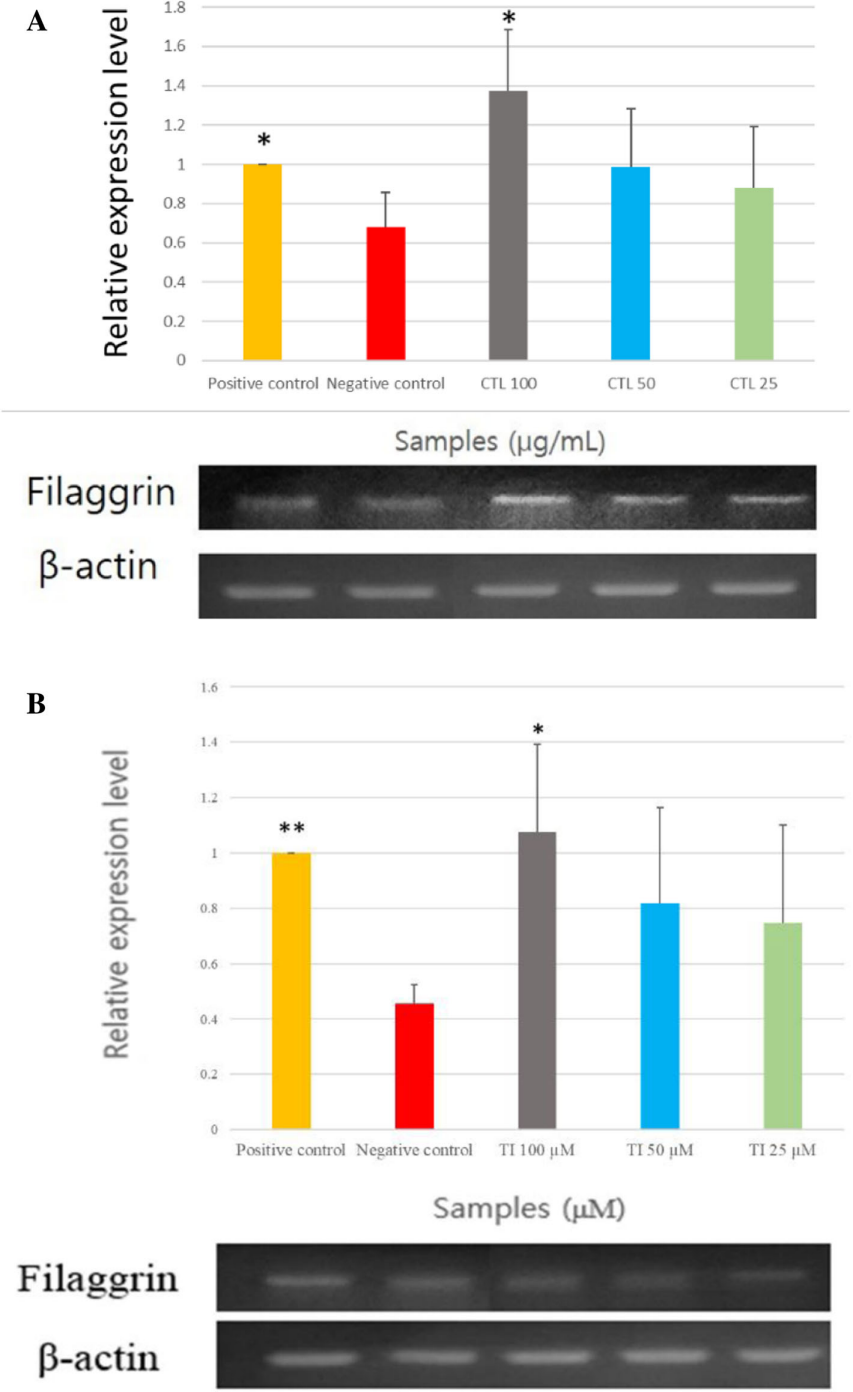
Relative filaggrin mRNA expression level upon treatment with (**a**): *Carpinus tschonoskii* leaf (CTL) extract and (**b**): tellimagrandin I (TI) normalized to β-actin. The results are expressed as mean ± standard deviation (SD) of triplicate experiments. (*n* = 3) **, *p* < 0.01; *, *p* < 0.05, comparison with negative control group

Relative filaggrin mRNA expression levels were evaluated based on the positive control, 1.5 mM Ca^2+^ treated group. Filaggrin mRNA expression levels appeared to be strongly induced upon treatment with the CTL extract compared with the positive control (Fig. 1). The filaggrin mRNA expression levels with CTL extracts were greater than those obtained with the positive control. At the compound level, filaggrin mRNA expression level showed potent increases with TI treatment compared with the positive control. The filaggrin mRNA expression level normalized to β-actin with 100 μM of IT was found to be similar to the positive control and greater than negative control.

#### 5α-reductase inhibitory activity

**Fig. 2:**
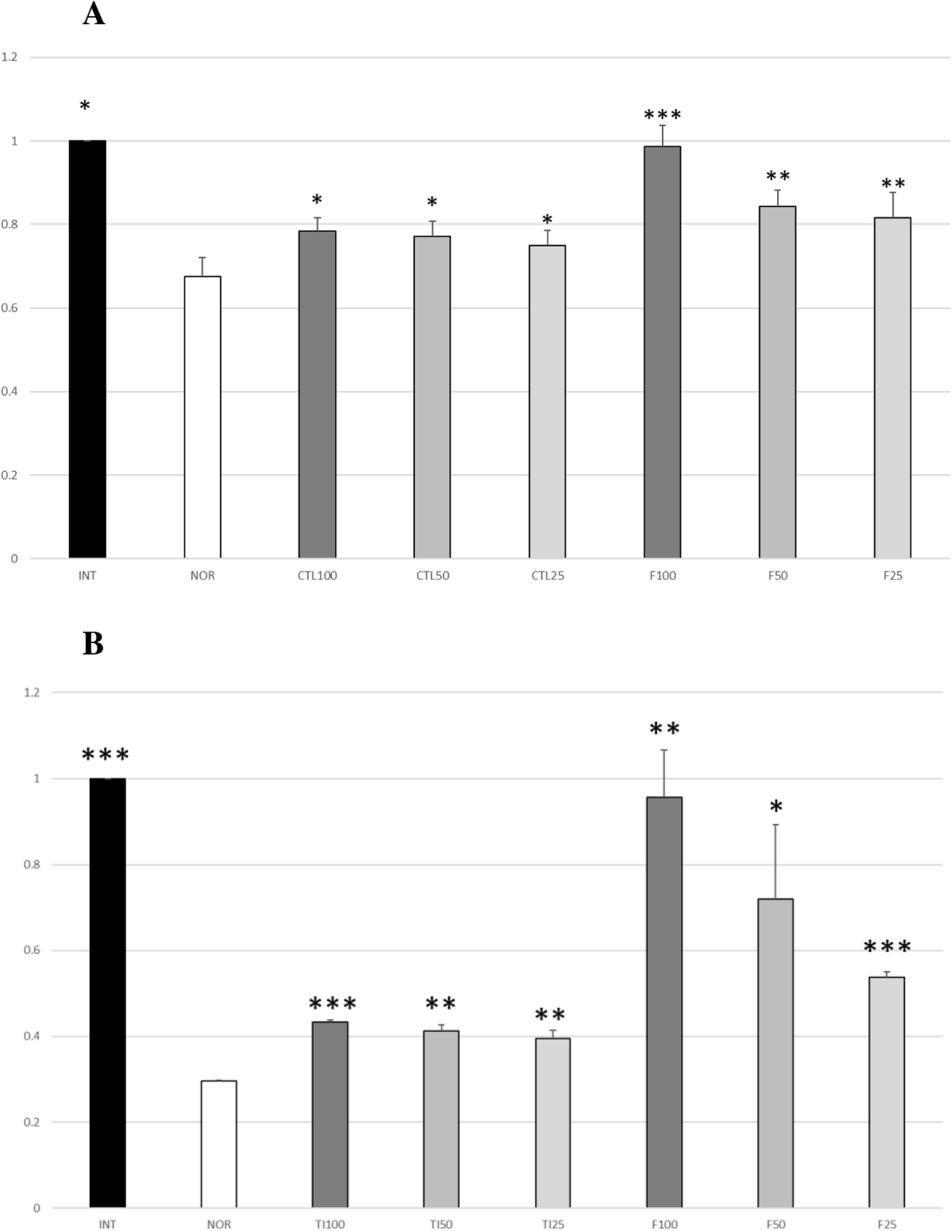
Relative testosterone level upon treatment with (**a**): *Carpinus tschonoskii* leaf (CTL) extract and (**b**): tellimagrandin I (TI). The results are expressed as mean ± standard deviation (SD) of triplicate experiments. (*n* = 3) ***, *p* < 0.001; **, *p* < 0.01; *, *p* < 0.05, comparison with normal control group

To evaluate the 5α-reductase inhibitory activity, the content of testosterone was determined through HPLC. The retention time of testosterone was 11.36 ± 0.1 min and was separated without interfering with other substances. Compared with the normal control, the sample-treated groups showed less decreased testosterone contents. Thus, CTL extract and TI suppressed the reaction of 5α-reductase with testosterone in a dose-dependent manner. As shown in Table 1 and Fig. 2, the CTL extract and TI showed 5α- reductase inhibitory activity.

## Discussion

ROS and NO plays an important role in the adjustment of the skin’s response to stimuli such as UV, heat, and infection. NO synthase 2 (NOS2) that generates NO is found to be expressed in inflammatory skin barrier diseases such as psoriasis [45, 46]. Ellagitannins possess hexahydroxydiphenyl (HHDP), which is the reason for the anti-oxidative activity of TI [47–49].

IL-6 remarkably upregulates the expression of MAP 17, which reduces filaggrin expression and downregulates CE formation, and activated IL-6 is an immune regulator produced by human keratinocytes [15, 16, 50–52]. IL-8 induces ROS, which causes damage to keratinocytes and stimulates production of inflammatory cytokines and lipid chemoattractants such as leukotriene B_4_ [11, 53]. In this experiments, CTL and TI showed potent anti-oxidative, anti-inflammatory and cytokine production inhibitory activities suggested that CTL and TI are good agents to protect skin and regulate the immune system. Comparing with other monomeric ellagitannins from other natural plants, TI showed potent anti-oxidative, anti-inflammatory and cytokine production, similar to the positive control, EGCG [47, 48].

CE is one of the most important components of barrier function and is a criterion to distinguish the late differentiation of keratinocytes. Correct formation of CE is important for skin barrier functions [5, 16]. Filaggrin plays an important role in the formation of CE. Filaggrin aggregates keratin filaments in corneocytes. It plays a role in the mechanical strength of the CE, and thus, is the essential factor of barrier function [54]. In addition, filaggrin is degraded to amino acids such as histidine and glutamine, which are respectively converted to urocanic acid and 2-pyrrolidone-5-carboxylic acid, which are important moisturizing factors [55]. Previously, it was reported that *Eucommia ulmoides* oliver (EU) showed CE and filaggrin enhance effect and CTL showed potent CE and filaggrin enhance effect comparing with 50 μg/mL of EU [44]. Therefore, CTL and TI increased CE and filaggrin protein level is a good way to develop skin barrier.

Androgen is reported to be a key factor in acne pathogenesis; especially, DHT in adulthood could induce balding, prostate growth, and sebaceous gland activity [56]. Furthermore, the activated sebaceous gland increases the production of sebum leading to blockage of the sebaceous gland duct, causing acne. The enzyme 5α-reductase metabolizes testosterone to DHT, but abnormally high 5α-reductase activity results in excessive DHT production [26]. .It is reported that caffeoyl derivatives from *Adina rubella* showed good 5α-reductase inhibitory activity [57]. In this experiment, CTL and TI inhibit androgen-induced sebum production by inhibiting the 5α-reductase reaction that produces DHT.

In conclusion, CTL and TI showed high activity as anti-AV endogenous factors, such as anti-oxidative and anti-inflammatory activities, cytokine inhibition, 5α-reductase inhibition, and exogenous factor activities, such as CE formation and increased filaggrin mRNA expression, to improve the skin barrier. Although TI which was isolated from CTL seemed to be potential for the application as new drug CTL extract also seem more economic value than TI for the extract contains other ellagitannins.

## Conclusion

CTL and TI showed potent anti-oxidative and anti-inflammatory activities with improved CE formation, increased filaggrin mRNA expression, and 5α-reductase inhibitory activity. These results suggest that CTL and TI might be plant-derived agent, promising in the treatment of acne vulgaris.

## Data Availability

The data that support the findings of this study are available from the corresponding author upon reasonable request.

## Abbreviations

AV: Acne vulgaris
CE: Cornified envelope
CT: *CARPINUS tschonoskii*
CTL: CT leaves
DHT: Dihydrotestosterone
DMEM: Dulbecco’s Modified Eagle Medium
DPPH: 1,1-diphenyl-2-picrylhydrazyl
DTT: Dithiothreitol
DTT: Thiazolyl blue tetrazolium bromide
EGCG: Epigallocatechin gallate
FBS: Fetal bovine serum
HHDP: Hexahydroxydiphenyl
IL-6: Interleukin-6
IL-8: Interleukin-8
L-NMMA: NG-Methyl-l-arginine acetate salt
LPS: Lipopolysaccharide
NADPH: Nicotinamide adenine dinucleotide phosphate
NO: Nitric oxide
NOS2: NO synthase 2
OD: Optical density
PBS: Phosphate buffered saline
RIA: Radioimmunoassay
ROS: Reactive oxygen species
SD: Sprague-Dawley
SDS: Sodium dodecyl sulfate
TEWL: Transepidermal water loss
TI: Tellimagrandin I
TNF- α: Tumor necrosis factor α
